# From parasite-induced immune activation to neuroinflammation and behavioral dysfunction: convergent mechanisms across protozoa and helminths: a review

**DOI:** 10.1186/s12974-026-03804-4

**Published:** 2026-04-17

**Authors:** Al- Shaimaa Mohsen Sadek, Reem Hamada Mahmoud

**Affiliations:** 1 Parasitology, Zoology and Entomology Department, Faculty of Science, Al-Azhar University, Nasr City, Cairo Egypt; 2 Under- graduate student, Zoology and Entomology Department, Faculty of Science, Al-Azhar University, Nasr City, Cairo Egypt

**Keywords:** Parasitic infections, Gut–brain axis, Neuroinflammation, Neuropsychiatric disorders, Microbiota dysbiosis, Cytokines, Neuromodulators, Epigenetics, Global mental health

## Abstract

**Background:**

Parasitic infections are increasingly linked to persistent neurological and psychiatric sequelae, yet the mechanistic routes from peripheral infection to altered brain function remain incompletely integrated across parasite groups. Emerging work suggests that parasites may influence the central nervous system indirectly through microbiota disruption and chronic immune activation, or directly through neurotropism, ultimately converging on neuroinflammation.

**Main body:**

Here, we synthesize human and experimental evidence that protozoan and helminth infections can remodel the microbiota–immune–brain axis and promote neuroinflammatory states associated with behavioral dysfunction. Across neurotropic (e.g., *Toxoplasma gondii*, *Plasmodium* spp., *Trypanosoma* spp.) and primarily intestinal parasites (e.g., *Enterobius vermicularis*, *Toxocara* spp., schistosomes, and *Taenia solium*–associated neurocysticercosis), convergent pathways include: (i) sustained peripheral cytokine production and immune-cell reprogramming; (ii) gut dysbiosis with increased microbial products and reduced short-chain fatty acids; (iii) increased intestinal permeability and enhanced immune-to-brain signaling; (iv) blood–brain barrier dysfunction and activation of microglia and astrocytes; and (v) downstream neurochemical and transcriptional remodeling, including perturbations in dopamine/serotonin/GABA-related signaling, indoleamine 2,3-dioxygenase–driven tryptophan catabolism, and infection-associated epigenetic and microRNA changes. Clinically, these cascades are associated with seizures, sleep disturbance, cognitive impairment, mood and anxiety symptoms, and under specific contexts psychosis-like phenotypes.

**Conclusions:**

We propose an integrative mechanistic model in which parasite-induced microbiota disturbance and chronic immune activation converge on glial activation and barrier dysfunction to shape brain circuitry and behavior. Defining shared neuroinflammatory nodes across parasitic diseases may reveal tractable biomarkers and host-directed therapeutic strategies to mitigate long-term neuropsychiatric risk.

## Introduction

Parasitic infections are among the most widespread chronic exposures in humans, particularly in low- and middle-income countries, yet their capacity to reshape host neurobiology through the microbiota gut brain axis has only recently come into focus [1]. The gut brain axis is now understood as a bidirectional, multisystem communication network in which the intestinal microbiota interact with the central nervous system (CNS) via immune, neuroendocrine, metabolic, and neural (especially vagal) pathways. Disturbance of this axis, most notably through gut dysbiosis and low-grade inflammation has been implicated in a wide range of neurological and psychiatric conditions, including depression, anxiety, schizophrenia, and neurodegenerative diseases [2]. To date, most research has concentrated on bacteria, diet, and antibiotics as primary drivers of dysbiosis, however, parasitic organisms such as protozoa and helminths represent a major but still underappreciated class of modulators of gut ecology, host immunity, and brain function [3].

It is increasingly recognized that intestinal parasites can alter gut microbiota composition and microbial metabolic outputs (e.g., short-chain fatty acid profiles) and exert immunomodulatory effects both directly and indirectly through microbiota-mediated pathways [4]. Also, helminths can increase bacterial richness and shift community structure, modifying short-chain fatty acid production, mucin expression, and epithelial barrier integrity, with downstream consequences for systemic immune tone and neuroinflammatory susceptibility [5].

Recent experimental and clinical evidence demonstrates that parasitic protozoa and helminths act within a parasite- microbiota- immune triad, in which each element continuously influences and reshapes the others as part of a dynamic ecological system [6].These interactions can generate either pro-inflammatory or regulatory immune states, influencing not only local intestinal pathology but also extraintestinal organs, including the CNS, through circulating cytokines, microbial metabolites, and neural signaling [7]. Several parasites acquired via the intestinal route, including those capable of neurotropism or strong neuroinflammation provide clinical proof of concept that infection can be associated with substantial neurological and psychiatric morbidity [8].

Peripheral infection can influence brain function even in the absence of direct pathogen invasion of the CNS, through coordinated immune to brain signaling pathways that include humoral mediators (e.g., circulating cytokines acting at the brain vasculature and circumventricular organs), neural afferent pathways, and secondary activation of glial cells within the CNS [9, 10]. These processes contribute to “sickness behavior” (e.g., fatigue, reduced motivation, social withdrawal, sleep disturbance, and impaired attention), which is initially adaptive during acute infection but may transition into persistent affective and cognitive symptoms when inflammatory signaling becomes chronic or dysregulated [9]. In addition, immune activation can alter neurotransmitter-relevant pathways (including tryptophan metabolism and glutamatergic signaling), synaptic plasticity, and neuroendocrine function, thereby creating biologically plausible links between chronic infection, inflammation, and mood/cognitive changes [9, 10].Within this broader framework, the gut microbiota- gut- brain axis offers an additional route by which parasitic infections may shape behavior through effects on intestinal inflammation, barrier function, microbial metabolites, and immune signaling [1, 11].

Helminths and protozoa often elicit distinct immune response profiles, and summarizing this contrast early provides essential context for interpreting later parasite-specific discussions of inflammation, microbiota–gut–brain signaling, blood brain barrier (BBB**)** integrity, and neuroinflammatory risk. Chronic helminth infections frequently induce a predominantly type 2/regulatory immune milieu (e.g., Th2 polarization with regulatory T-cell activity and IL-10–linked immunomodulation), which can dampen bystander inflammation and reshape mucosal immune tone [12]. In contrast, control of many intracellular protozoa relies more heavily on type 1 pro-inflammatory immunity, typically initiated by IL-12 and mediated by IFN-γ (often with TNF-α), which is central to host resistance but can also increase exposure to pro-inflammatory cytokines relevant to immunopathology [13].

Globally, several parasitic infections are associated with substantial neurological and neuropsychiatric morbidity. Neurocysticercosis (NCC), caused by the larval stage of *Taenia solium*, is a major contributor to acquired epilepsy in endemic regions, with systematic evidence showing a substantial frequency of NCC among people with epilepsy across Latin America, sub-Saharan Africa, and parts of Asia [14]. In regions where *T. solium* transmission is endemic, neurocysticercosis is frequently identified among individuals with late-onset seizures and is strongly implicated as an important contributor to epilepsy burden; consistent with this, controlled clinical trial data demonstrate that antiparasitic therapy reduces parasite burden and can reduce specific seizure outcomes in patients with viable parenchymal cysts [15–17].

Several primary studies have proposed that *T. gondii* can influence dopamine metabolism or dopamine-associated behaviors in experimental models, including reports of increased dopamine release in infected dopaminergic cells and mechanistic hypotheses involving parasite-encoded aromatic amino acid hydroxylase/tyrosine activity; however, other primary work has challenged the reproducibility and generality of a uniform dopamine-elevation phenotype [18, 19].

Despite all of this consistent evidence, current conceptualizations of the microbiota- gut- brain axis rarely integrate parasitology in a systematic way. Many reviews treat infection as a generic inflammatory trigger or focus narrowly on bacterial pathogens, without accounting for the distinctive life cycles, tissue tropisms, and immunoregulatory strategies of intestinal parasites [20]. On the other hand, classical parasitology has usually attributed neurological symptoms such as seizures in neurocysticercosis or behavioral changes in chronic toxoplasmosis to direct effects within the CNS, giving far less attention to the possibility that gut colonization, parasite-driven dysbiosis, and systemic immune changes may also influence these outcomes through the gut–brain axis [21].

From all, intestinal parasites may act not only as occasional neurotropic pathogens, but also as long-term influences of the gut environment and the immune system, with downstream effects on brain function and behavior. In this review, we introduce the concept of parasite-induced modulation of the gut–brain axis as a framework that connects parasitology, microbiome research, and neuropsychiatry.

Drawing on examples including *Taenia solium*, *Toxoplasma gondii*, trypanosomes, *Enterobius vermicularis*, *Toxocara* spp., *Schistosoma* spp., *Onchocerca volvulus*, and *Plasmodium falciparum*, we summarize emerging evidence that intestinal infection and the dysbiosis that follows can affect neurodevelopment, cognition, and mental health. These impacts may arise through inflammatory responses, alterations in the blood–brain barrier, microbially derived metabolites, or parasite-produced molecules that interact with neural pathways.

By bringing these observations together within a shared mechanistic framework, our goal is to highlight new ways in which parasitic diseases may contribute to the global burden of neuropsychiatric disorders and to point toward multi-omics, systems-level research approaches that can clarify causal pathways and guide future therapeutic and preventive strategies.

### Mechanistic insights

#### Neuroinflammatory pathways and cytokine networks

Parasitic infections can engage innate immune pathways in the CNS, but the specific receptors and mediators involved differ by pathogen, host species, and infection route. In *T. gondii* infection, CNS immune activation involves multiple pattern-recognition and inflammasome-linked pathways; however, evidence supporting a dominant, infection-induced TLR2-dependent mechanism in the brain should be interpreted cautiously. In a murine latent toxoplasmosis model, TLR2 deficiency altered cerebral immune readouts and behavioral measures without demonstrating a clear infection-specific, TLR2-dependent interaction, indicating that TLR2-linked effects may reflect baseline host differences rather than direct parasite-driven induction [22]. In addition, during oral infection, innate signaling attributed to TLR2/4 can be amplified indirectly through sensing of gut commensals that shape host responses against *T. gondii* [23]. In contrast, experimental cerebral malaria models show robust neuroinflammatory cytokine induction in brain tissue, including increased IL-1β and TNF-α, consistent with strong inflammatory activation during infection [23].

Notably, some chronic helminth infections can attenuate neuroinflammatory tone by releasing immunomodulatory products that interfere with canonical microglial innate signaling upstream of NF-κB, thereby limiting key activation steps required for NF-κB dependent transcription and reducing downstream pro-inflammatory outputs such as IL-6 and TNF-α [24]. Importantly, inflammation itself is tightly linked to behavioral and cognitive phenotypes: TNF-α was originally identified as “cachectin” based on its cachexia-inducing effects, and IL-1/TNF family signaling has long been recognized as capable of driving sickness-related behavioral programs [25]. Thus, across parasite systems, infections may either promote or suppress inflammatory pathways associated with neurobehavioral change; in humans, higher IL-6 and TNF-α levels have been associated with more severe depressive symptoms [26], and in experimental models where parasite infection is accompanied by increased brain IL-1β and TNF-α, animals commonly exhibit sickness behavior like phenotypes including anhedonia-like and anxiety-like behavior [23].

Epidemiological findings on latent *T. gondii* seropositivity and common mental disorders are mixed. Some population studies report associations with specific outcomes such as generalized anxiety disorder and depressive symptom measures, whereas other large cohorts find no association with depressive or anxiety disorder diagnoses after adjustment. Overall, the literature is largely observational and cross-sectional; therefore, causal direction and effect size remain uncertain [27].

Parasitic infection can provoke neuroimmune signaling that may interact with neurotransmitter and stress-regulation pathways. In animal models, infection has been associated with diverse behavioral changes, but the direction and specificity of anxiety or depression-like phenotypes vary by host, parasite strain, and experimental context. Human evidence linking latent parasitic infection to depression or anxiety is suggestive in some studies but inconsistent overall [10].

#### Parasite-derived neuromodulators

Many parasites can directly interfere with brain signaling by producing substances that mimic the body’s own neurotransmitters. *T. gondii* encodes aromatic amino acid hydroxylases (AAH1/AAH2) that can catalyze L-DOPA production and were proposed to influence host dopamine pathways. However, multiple genetic studies report that deletion of AAH2 does not abolish infection-associated behavioral phenotypes or dopamine-linked neurochemical changes, and some work does not support widespread host dopamine elevation during infection. Importantly, AAH genes appear to contribute to parasite fitness during the sexual cycle in cats, suggesting alternative (or additional) functions related to transmission biology and/or structural processes. Thus, while dopamine-pathway interactions remain an active hypothesis, current evidence does not justify treating parasite AAH enzymes as definitive proof of host neuromodulation [19].

#### Epigenetic and microRNA alterations

Beyond neurotransmitter and immune effects, chronic parasitic infection can be associated with changes in neuronal gene regulation, including epigenetic modification and microRNA (miRNA) dysregulation. Experimental evidence indicates that chronic *T. gondii* infection may reduce central noradrenergic signaling, including decreased expression of dopamine beta-hydroxylase (DBH), the enzyme required for norepinephrine biosynthesis [28]. In one model, infection induced host-derived extracellular vesicles (TINEVs) were reported to downregulate DBH expression and induce hypermethylation upstream of the DBH locus, including in enriched neuronal populations from chronically infected mice [29]. These findings support an EV-linked, infection-associated pathway that may alter catecholaminergic signaling, including in bystander cells.

In parallel, in vivo profiling studies have shown that *T. gondii* infection is accompanied by altered brain miRNA expression during both acute and chronic stages [30, 31]. The dysregulated miRNAs are enriched for targets involved in synaptic function, immune signaling, oxidative stress, cell survival, and stress-response pathways, including the unfolded protein response [32]. For example, immune regulatory miRNAs such as miR-146a and miR-155 were reported to be upregulated in infected brain tissue, suggesting potential effects on neuroinflammatory signaling and neuron–glia communication [33].

However, these molecular findings should be interpreted cautiously. In vivo comparisons between infected and uninfected animals may capture both direct infection effects and secondary changes related to the broader neuroinflammatory milieu, making mechanistic attribution difficult without additional controls that isolate inflammation from infection. Accordingly, the current evidence supports infection associated molecular remodeling of neuronal regulatory pathways (including DBH repression and miRNA shifts), but the extent to which these changes directly drive persistent cognitive, affective, or behavioral phenotypes remains to be established through studies with direct behavioral and clinical endpoints [29].

### Host–parasite pathways shaping brain and behavior

#### The infection immunity brain axis

Parasitic infections can profoundly influence host brain function through neuroimmune pathways. Upon infection, the innate immune system detects parasite molecules using pattern recognition receptors such as TLRs and nucleotide-binding oligomerization domain-like receptors (NODs) on macrophages, dendritic cells, and glial cells. This detection initiates MyD88-dependent signaling cascades that lead to the release of large quantities of proinflammatory cytokines, including IL-1β, IL-6, IL-12, TNF-α, and IFN-γ, as well as chemokines [34–36].

Protozoan infections typically induce a Th1-dominated immune response, marked by IFN-γ, IL-12, CD8⁺ T cells, and activated macrophages. In contrast, helminth infections favor a Th2 response, characterized by IL-4, IL-5, IL-13, elevated IgE, and eosinophilia [37]. These cytokine profiles activate brain endothelial cells, increasing expression of ICAM-1 and VCAM-1 and facilitating leukocyte adhesion and migration across the blood brain barrier (BBB) [36]. In the central nervous system, microglia and astrocytes become activated and produce additional proinflammatory mediators such as IL-6, CXCL10, GM-CSF, nitric oxide (NO), and reactive oxygen species (ROS), which collectively drive neuroinflammation and contribute to neuronal injury [38, 39].

A well characterized consequence of neuroimmune activation is sickness behavior, a conserved syndrome including fever, lethargy, anorexia, reduced exploration, and social withdrawal mediated by pro-inflammatory cytokines (notably IL-1β, IL-6, and TNF-α) acting on central circuits that regulate motivation and energy balance [40]. During acute infection, these behaviors are generally considered adaptive because they conserve energy and can reduce risk taking and exposure. Consistent with this framework, peripheral immune stimulation (e.g., lipopolysaccharide) or administration of pro-inflammatory cytokines in animal models reproduces key components of sickness behavior [40, 41]. Against this host protective baseline, host manipulation theory proposes that in some parasite life cycles, selection may favor parasite induced suppression or redirection of sickness associated avoidance (e.g., maintaining exploration, mating, or activity) when doing so increases transmission opportunities [42]. Importantly, when neuroinflammation becomes chronic or dysregulated, downstream phenotypes often categorized as “pathological” including depression-like behavior, anhedonia, and cognitive impairment may also influence transmission relevant outcomes by altering threat assessment, vigilance, and avoidance, potentially increasing vulnerability to predation or other routes of parasite acquisition [43–45].

IFN-γ, a key cytokine in host control of *T. gondii*, can induce IDO-mediated tryptophan catabolism and shift tryptophan metabolism toward the kynurenine pathway (elevating Kyn/Trp ratios in plasma and brain during acute infection), which may secondarily alter serotonergic turnover; however, behavioural and neurotransmitter outcomes differ by infection stage and model [46]. TNF-α and IL-1β can also alter synaptic transmission by modifying glutamate receptor function, potentially contributing to fatigue, brain fog, and attentional deficits [47]. Some parasites have been proposed to influence host neurochemistry, but the strength of evidence and mechanistic attribution vary by pathway and experimental context. *T. gondii* encodes aromatic amino acid hydroxylases (AAH1/AAH2) capable of generating L-DOPA [48], and dopamine changes have been reported in some in vitro and in vivo models; however, in vivo genetic disruption studies indicate that parasite AAH activity is not required for measurable host dopamine changes or dopamine-linked behavioral phenotypes, arguing against presenting dopamine modulation as a settled mechanism [19, 49]. Accordingly, observations such as immunostaining of tissue cysts with dopamine-reactive antibodies should be interpreted cautiously and do not, on their own, demonstrate host dopamine manipulation.

In contrast, there is direct experimental evidence that chronic infection can be accompanied by disrupted glutamatergic regulation in vivo: microdialysis studies in infected mice identified increased extracellular glutamate in frontal cortex in association with reduced expression of the astrocytic glutamate transporter GLT-1 [50].

**Fig. 1:**
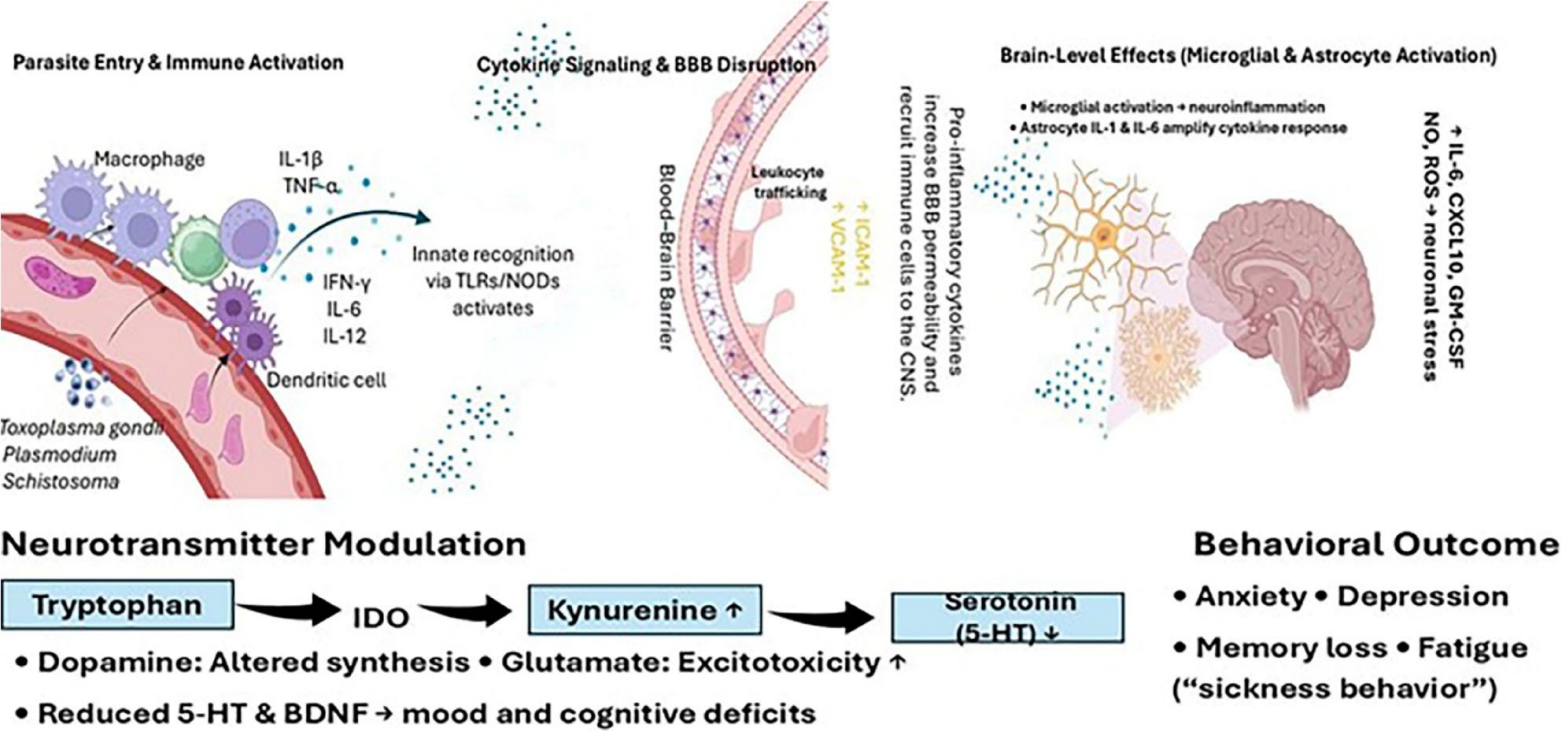
The infection immunity brain axis: linking parasitic infections to neuroinflammation and behavioral changes

In summary, the infection–immunity–brain axis (often framed as a parasite–immune–neurotransmitter triad) describes how parasitic infections can alter brain function through coordinated peripheral and central immune signaling. Pattern recognition driven cytokine release can activate CNS-resident glia and BBB endothelium, enabling neuroinflammatory signaling within the brain and shifting neurotransmitter related metabolism, most notably through interferon linked engagement of the tryptophan–kynurenine pathway. Downstream kynurenine metabolites may contribute to neurobehavioral phenotypes: kynurenic acid (KYNA) is generally viewed as relatively neuroprotective, whereas quinolinic acid (QUIN), an NMDA receptor agonist, can promote excitotoxicity and amplify neuroinflammation, thereby plausibly contributing to cognitive impairment, attentional deficits, and “brain fog”-like symptoms. Proposed direct neuromodulatory effects of parasites (e.g., dopamine-related changes) remain model dependent and should be interpreted cautiously. Clinically and experimentally, acute infections may present with overt neuroinflammatory outcomes (e.g., encephalopathy or seizures in severe contexts), whereas chronic infections may be associated with subtler fatigue, cognition, or mood related changes depending on parasite species, host factors, and infection stage. As illustrated in Fig. 1, parasitic infections initiate an immune cascade that affects neurotransmitter metabolism and neural circuitry, ultimately contributing to observable changes in behavior and mood.

#### The microbiota-gut-brain axis in parasitic infections

Intestinal parasitic infections influence much more than the gut itself, particularly through their effects on the microbiota–gut–brain axis. This bidirectional communication system integrates the gut microbiome, intestinal barrier, and central nervous system via neural (primarily vagal), hormonal, and immune pathways [51]. As a result, disruptions in the gut microbial ecosystem can influence cognitive function, mood regulation, and emotional behavior.

In protozoan infection, the intestinal phenotype can differ substantially from helminth-driven remodeling. Oral *T. gondii* infection in experimental models has been linked to inflammatory intestinal pathology with microbiota destabilization and expansion of inflammation-associated taxa (e.g., Enterobacteriaceae/Proteobacteria), and several in vivo studies indicate that host responses attributed to “parasite sensing” can be strongly shaped by commensal recognition and barrier disruption during infection [52]. By contrast, helminth infections more commonly bias mucosal immunity toward type 2/regulatory programs (Th2/Treg), altering mucus, epithelial turnover, and metabolite landscapes in ways that can shift microbial community structure and function often in a species and context-dependent manner [53, 54].

Parasitic infections frequently induce gut microbiota dysbiosis, with significant alterations in bacterial composition observed in helminth-infected hosts. For instance, mice infected with *Heligmosomoides polygyrus* exhibit substantial microbial shifts, including increases in *Bacteroidetes* and reductions in *Firmicutes*, notably *Lactobacillus* species [55]. Similar findings have been reported in pigs experimentally infected with *Trichuris suis*, where approximately 13% of bacterial genera were altered, including marked reductions in *Fibrobacter* and *Ruminococcus* populations [56]. These changes suggest that the presence of parasites frequently disrupts the microbial balance of the gut. In most cases, such community shifts are driven by immune responses and changes to the intestinal environment triggered by the parasites themselves.

Helminths promote a Th2/regulatory immune phenotype characterized by elevated IL-4, IL-13, and IL-10, which modulates gut physiology by increasing mucus secretion, altering antimicrobial peptide profiles, and influencing IgA production, all of which create favorable conditions for specific microbial taxa [57]. In the case of *Heligmosomoides polygyrus*, the observed microbiota changes were found to be dependent on the host s Th2 immune response [55].

Additionally, parasites can directly modulate the microbiome through secreted molecules. Helminth-derived excretory secretory products have been shown to induce tolerogenic dendritic cells and expand regulatory T-cell populations, both of which influence microbial community structure [58]. In a mouse model of extraintestinal *Echinococcus* infection, this interaction led to an expansion of butyrate-producing *Lactobacillus* in the feces, accompanied by an increase in colonic regulatory T cells [59]. These findings highlight a dynamic tripartite network in which the parasite, host immune response, and gut microbiota interact continuously to shape intestinal homeostasis [55, 59]. Depending on the context, this modulation can lead to either increased microbial diversity (as seen in some helminth infections) or selective overgrowth and depletion patterns commonly described as dysbiosis [56].

These microbiota alterations carry important neurobehavioral consequences. In children, heavy parasite burdens are associated with diminished performance in learning, memory, and IQ assessments [60]. While malnutrition and iron deficiency anemia are well established contributors to these cognitive impairments, gut dysbiosis is now recognized as a potential co-factor. The emerging concept of the helminth microbiota CNS axis proposes that helminth infections disrupt microbial communities, thereby initiating downstream neuroimmune and metabolic signals that impair cognitive development. Supporting this model, studies in animal systems have shown that even in the absence of parasitic infection, experimental disruption of the gut microbiota via broad spectrum antibiotics or unhealthy diets can impair learning and memory [61–63]. Despite these mechanistic links, large scale deworming trials in children have yielded mixed and largely inconclusive results regarding cognitive improvement. A recent Cochrane systematic review concluded that routine anthelmintic therapy in endemic regions is unlikely to produce measurable gains in cognitive outcomes among school-aged children [64]. This suggests that helminths may not exert direct neurotoxic effects but instead contribute to cognitive impairment indirectly through chronic intestinal inflammation, impaired nutrient absorption, and shifts in microbial composition.

Further support for this indirect model comes from both animal experiments and observational studies in humans, which reinforce the idea that parasite induced dysbiosis can affect cognition through neuroimmune and metabolic signaling [61, 63]. Chronic enteric inflammation related to parasitic infection may further compromise nutrient availability and interfere with neurodevelopmental processes such as neural growth and synaptic plasticity, increasing the risk of long-term cognitive deficits [63, 65].

The gut microbiota also serves as a major source of neuromodulatory molecules, including short chain fatty acids (SCFAs) and serotonin precursors. Parasite-induced dysbiosis can therefore impact the availability of these compounds and affect mood and anxiety regulation [66]. One notable example involves *Blastocystis*, a common gut protist that possesses a gene for tryptophanase (BhTnaA), enabling it to convert microbial indoles into excess tryptophan. In a recent mouse study, *Blastocystis*-colonized animals displayed elevated colonic tryptophan and serotonin levels, accompanied by significantly heightened anxiety-like behavior [67]. This effect is thought to be mediated via the vagus nerve, as enterochromaffin-derived serotonin activates vagal afferent signaling.

On the other hand, dysbiosis may increase populations of lipopolysaccharide (LPS)-producing bacteria. During episodes of gut inflammation, elevated LPS can breach a compromised intestinal barrier (leaky gut), enter systemic circulation, and trigger peripheral cytokine release. These systemic inflammatory signals can cross into the brain and promote neuroinflammation, a pathway linked to mood disorders such as depression and anxiety [51].

Interestingly, parasite induced shifts in the microbiota are not uniformly negative. Several helminths encourage the proliferation of SCFA-producing bacterial taxa, a change that can support gut health and dampen inflammation. For instance, *H. polygyrus* infection has been associated with increased fecal concentrations of SCFAs, such as butyrate [59]. These metabolites are known to support intestinal barrier integrity, modulate microglial activation, and enhance regulatory T-cell responses that collectively reduce inflammation and support brain health.

Although the microbiota–gut–brain axis provides a compelling mechanistic framework, human evidence linking parasitic infection to specific microbial “signatures” remains heterogeneous and should be interpreted cautiously. Large population-based analyses indicate that geography and associated environmental variation can explain substantial proportions of gut microbiome variance and can undermine cross region reproducibility of microbiota host associations, even within a single country [68]. Consistent with this, helminth–microbiome relationships in humans appear context dependent: metagenomic field data show that helminth-associated microbiota effects can be village dependent and that anthelmintic drugs (e.g., albendazole) may themselves exert substantial and potentially confounding impacts on microbial communities, which may contribute to discrepancies across studies [69]. Intervention studies further suggest that baseline helminth infection may not always produce large, reproducible shifts in overall diversity, and that post deworming changes can be modest and partially attributable to treatment effects even in initially uninfected individuals [70]. Accordingly, rather than implying a conserved parasite-associated microbiome signature, a more conservative interpretation is that parasite exposure can modulate host–microbiome–immune interactions in a setting and species dependent manner, and that robust inference will require longitudinal, multi-site designs and improved reporting to address known reproducibility challenges in human microbiome research [71].

**Fig. 2:**
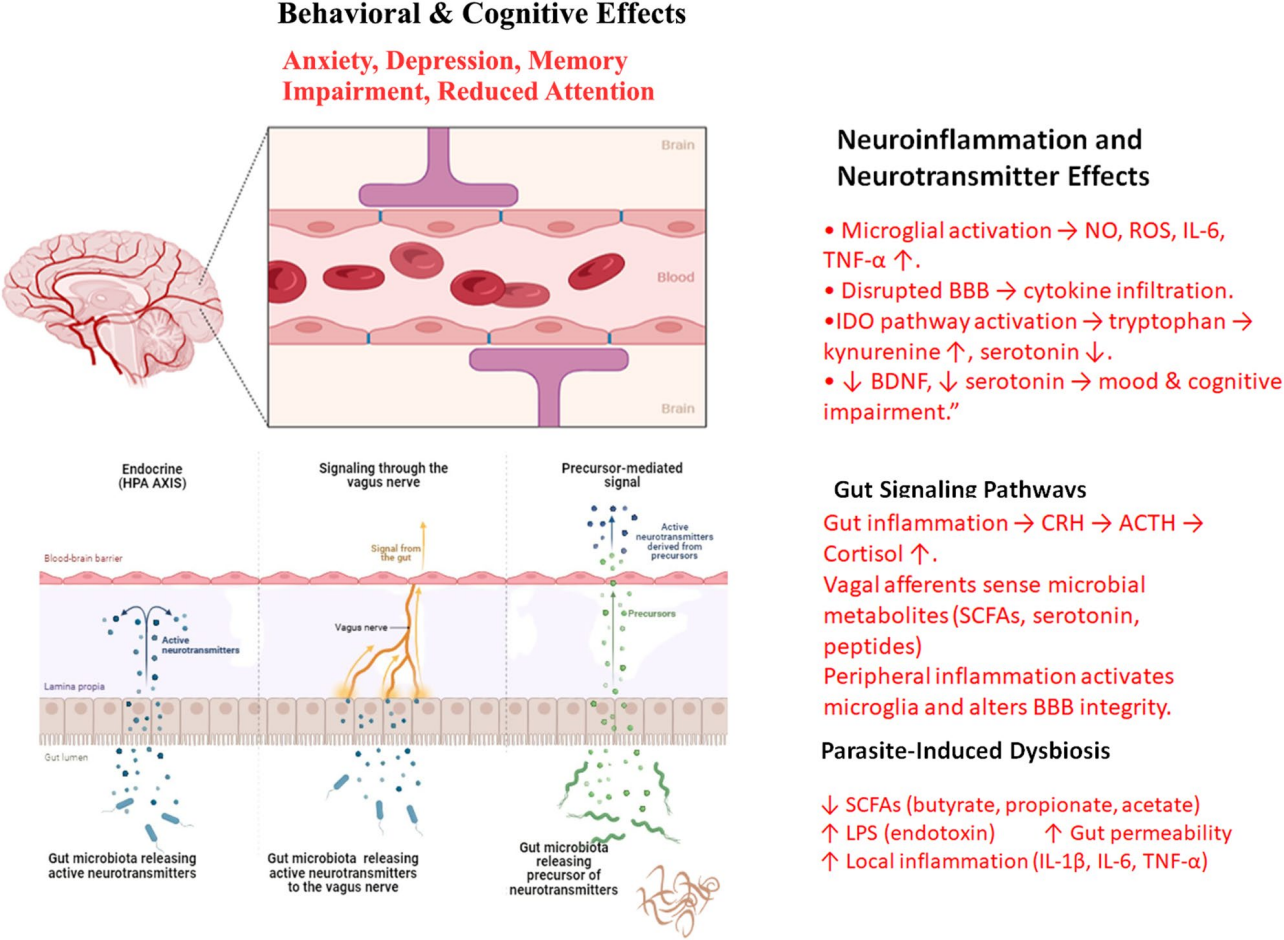
Mechanistic overview of the microbiota–gut–brain axis in parasite-induced neuroinflammation and behavioral dysfunction

In conclusion, the microbiota gut brain axis offers a compelling framework for understanding how intestinal parasites can influence the central nervous system without directly invading it. By reshaping the abundance and metabolic function of gut commensals, parasites alter the production of key neurometabolites (e.g., SCFAs, tryptophan, serotonin) and immune signals that influence brain. Although the parasites themselves remain confined to the gut, the physiological ripple effects they generate can reach the brain, modulating neural function, behavior, and emotion. Figure 2 illustrates how parasite-induced dysbiosis disrupts communication between the gut and brain via hormonal, immune, and microbial signaling pathways.

### Emerging links between parasites and brain function

#### *Taenia solium* and neurocysticercosis

Neurocysticercosis (NCC), caused by the larval stage (cysticerci) of the tapeworm *Taenia solium*, is a major neuroinfectious condition that can lead to long-term neurological and neuropsychiatric morbidity in endemic settings [72, 73]. Humans are typically accidental intermediate hosts of *T. solium* when they ingest parasite eggs via fecal- oral contamination; the released oncospheres disseminate and develop into larval cysticerci in tissues, including the central nervous system, causing neurocysticercosis and its major clinical sequelae [74]. The global burden remains substantial, with approximately 50 million individuals estimated to be infected and around 50,000 deaths annually, with the highest burden in regions where *T. solium* is endemic, particularly parts of Latin America, Africa, and Asia [75].

NCC is widely regarded as the most common human neuroparasitic infection and a leading cause of acquired epilepsy and other neurological morbidity in these regions. Mechanistically, NCC arises when humans ingest *T. solium* eggs via fecal-contaminated food or related fecal–oral exposure routes, after which larvae develop and may lodge in the CNS; symptom expression is strongly shaped by cyst number, size, anatomical location, and host immune responsiveness. Once cysticerci localize to the CNS, they elicit local inflammatory responses, and during degeneration they can disrupt blood–brain barrier integrity, facilitating immune-cell infiltration into brain tissue; consequently, clinical manifestations often reflect immune-mediated inflammation and raised intracranial pressure rather than direct mechanical injury alone [73].

Clinically, patients may present with seizures and persistent headaches as well as diverse neuropsychiatric outcomes including cognitive deficits, confusion, dementia, depression, anxiety, hallucinations, and personality or behavioral changes, with lesion location (e.g., frontal lobe involvement) plausibly contributing to executive dysfunction, apathy, impulsivity, or personality change in some cases [73]. Thus, NCC provides strong clinical proof-of-concept that parasitic infection can be associated with substantial neuropsychiatric and cognitive morbidity through direct CNS localization with secondary neuroinflammation, although synthesis across studies is limited by heterogeneity in cyst stage, lesion burden and distribution, and variability in standardized psychiatric assessment across settings [72, 73].

#### Toxoplasmosis

Toxoplasmosis is caused by the neurotropic protozoan *Toxoplasma gondii* and is of major clinical relevance because it can cause congenital infection and severe neurological complications, including encephalitis, chorioretinitis, and fetal brain damage, particularly in immunocompromised individuals, and has also been linked to neuropsychiatric manifestations [18, 76–79]. However, evidence increasingly suggests that even in immunocompetent hosts, chronic/latent infection may be associated with mild but clinically relevant neuropsychiatric effects [80]. Epidemiological evidence primarily from case control studies and meta-analyses suggests that *Toxoplasma* IgG seropositivity is more frequent among individuals with schizophrenia than among controls; however, these associations are modest, heterogeneous, and do not imply causality or that regions with high background seroprevalence necessarily have higher population rates of schizophrenia. Evidence linking latent toxoplasmosis to mood/anxiety outcomes and behavioral/personality traits is mixed and should be interpreted cautiously [81–83]. The proposed neurobehavioral effects of *T. gondii* are considered in the context of chronic infection/latency, where persistent host–parasite interactions may sustain low-grade immune activation and influence gut and brain signaling pathways, rather than only acute severe disease presentations [18, 59, 84].

In humans, latent toxoplasmosis has been associated with increased risk of schizophrenia and affective/anxiety disorders, as well as altered personality traits and risk-taking behavior [85, 86]. Severe CNS outcomes (e.g., encephalitis) are classically observed in immunocompromised hosts, while subtler neuropsychiatric associations have been reported in immunocompetent populations [80, 81].

Mechanistic models increasingly emphasize immune activation and gut–brain pathways. *T. gondii* infection can trigger gut inflammation and dysbiosis factors with plausible relevance to CNS function via the gut–brain axis [84, 86]. In rodent models, acute *T. gondii* infection can trigger pronounced small intestinal inflammation with concomitant shifts in gut microbial composition. In a chronic infection model, behavioral readouts were characterized primarily by reduced exploratory activity (which may overlap with sickness-like behavior), and antibiotic mediated microbiota depletion attenuated these effects; conversely, fecal microbiota transfer from infected donors exacerbated reduced exploration in recipients, supporting a microbiota contributory component without implying a uniquely anxiety specific phenotype [87].

In animal models, anxiety-stress-related phenotypes are commonly evaluated using the open field test (OFT) and elevated plus maze (EPM), while behavioral despair is often assessed with the forced swim test (FST) and tail suspension test (TST). In a chronic *T. gondii* mouse model, infected animals showed reduced open-arm exploration in the EPM and reduced center exploration in the OFT; however, these readouts should be described as reduced exploratory behavior, because overlap with sickness-like behavior cannot be excluded. In the same model, the authors reported lower 5-hydroxytryptamine, amygdalar microglial activation, and increased pro-inflammatory cytokine expression, supporting a link between altered exploration and neuroimmune/neurotransmitter changes [87].

Beyond behavioral results, *T. gondii* has been implicated in neurotransmitter-relevant pathways: the parasite encodes aromatic amino acid hydroxylases (AAH1/AAH2) capable of producing L-DOPA, a precursor of catecholamines; however, experimental work indicates these enzymes are important for parasite biology/transmission and do not provide definitive evidence that parasite AAH activity universally elevates host brain dopamine across models [19, 88]. Additional reports describe potential effects on glutamatergic and GABAergic signaling, but mechanistic interpretation should remain cautious. Importantly, claims that dopamine receptor targeting antipsychotics “reverse” neuropsychiatric like abnormalities in infected rodents should be avoided. Classic pharmacological studies more specifically show attenuation of *T. gondii* associated predator-odor aversion changes with haloperidol and anti-protozoal treatment, and the mechanism may include antiparasitic activity in addition to neuromodulatory effects [89].

Overall, human evidence for neuropsychiatric associations is largely epidemiological/clinical and therefore supports association rather than causality, whereas experimental models provide mechanistic support linking chronic infection to changes in exploration, neuroinflammation (microglia/cytokines), and neurotransmitter-related measures, with substantial dependence on host background, infection stage, and model system [81, 87].

#### American trypanosomiasis / chagas disease (*Trypanosoma cruzi*)

American trypanosomiasis (Chagas disease), caused by *Trypanosoma cruzi*, differs mechanistically from African trypanosomiasis in that chronic infection primarily affects the heart and gastrointestinal tract and is not typically classified as neurotropic, despite documented neurological and cognitive sequelae in some patients [90]. Transmission occurs mainly through triatomine (“kissing bug”) feces, and the chronic disease phase is characterized by cardiomyopathy and gastrointestinal pathology, including enteric nervous system damage that contributes to megaesophagus and megacolon, providing a biologically relevant gut/enteric context for indirect organ-axis effects on brain function [91]. Human studies have reported cognitive impairments, particularly affecting memory and executive function, in chronic Chagas patients, including reports in individuals without overt cardiac pathology, suggesting that brain effects may arise through indirect pathways rather than routine CNS parasitism [92]. Proposed mechanisms emphasize chronic systemic immune activation and neuroendocrine dysregulation, as chronic *T. cruzi* infection has been associated with elevated proinflammatory cytokines and stress hormones that could plausibly disrupt brain function via immune-to-brain signaling pathways [92]. Direct CNS invasion remains relevant but uncommon, occurring mainly in acute disease or immuno-compromised individuals where meningoencephalitis can develop. Overall, evidence supporting indirect cognitive effects in chronic Chagas disease is moderate and biologically plausible, but it is constrained by heterogeneity in clinical disease severity, potential confounding, and a limited number of longitudinal mechanistic studies linking immune biomarkers to neurocognitive outcomes over time [92].

#### African trypanosomiasis (*Trypanosoma brucei*)

African trypanosomiasis (“sleeping sickness”), caused by *Trypanosoma brucei*, is a major cause of neurological and neuropsychiatric morbidity in endemic areas [90]. Transmitted by the tsetse fly, infection typically progresses from an early hemolymphatic phase to a late encephalitic stage after parasites cross the blood–brain barrier and establish CNS involvement, which represents the key mechanistic transition for brain-related outcomes [73, 93]. Clinically, early-stage disease presents with nonspecific systemic symptoms (e.g., intermittent fever, lymphadenopathy, headache, fatigue), whereas late-stage disease is characterized by prominent sleep–wake disruption (daytime somnolence, insomnia, reversal of the sleep cycle), cognitive dysfunction, apathy, and personality change that may progress to severe neuropsychiatric features including irritability, hallucinations, confusion, manic symptoms, aggression, and suicidal ideation; neurological signs such as tremor, ataxia, and peripheral neuropathy may also occur, consistent with diffuse CNS involvement [73, 84, 93, 94]. Pathogenesis in the encephalitic stage is strongly linked to CNS inflammation: parasite proliferation in cerebrospinal fluid and brain parenchyma is associated with activation of astrocytes and microglia and a proinflammatory cytokine cascade, and elevated IL-6 and IL-10 levels in CSF have been reported as biomarkers of ongoing neuroinflammation with plausible relevance to sleep disturbance and cognitive decline [95]. In addition to immune-mediated injury, parasite molecules including variant surface glycoproteins have been proposed to contribute to endothelial and neuronal damage, potentially exacerbating neuropathology [96]. Overall, the evidence base for CNS involvement and neuroinflammation in late-stage African trypanosomiasis is strong due to the well-defined clinical staging and supportive CSF biomarker findings, although outcome heterogeneity by stage and the limited use of standardized psychiatric phenotyping remain important constraints for synthesis across studies [73, 93–95].

#### *Enterobius vermicularis* (pinworm)

*Enterobius vermicularis* (pinworm) is typically regarded as a common, mild helminth infection of childhood, yet recent evidence suggests it may be associated with measurable mental health and behavioral disturbances in pediatric populations. In a nationwide cohort study from Taiwan, children diagnosed with enterobiasis showed significantly higher odds of anxiety disorders, depressive disorders, and sleep disturbances compared with uninfected peers, indicating a clinically relevant association at the population level [97].

Clinically, case reports and routine observations describe chronic insomnia, irritability, and mood instability in children with heavy infestations, and the most widely supported mechanistic explanation is sleep disruption secondary to intense nocturnal perianal pruritus caused by gravid females depositing eggs at night; sustained sleep fragmentation may contribute to daytime fatigue, emotional dysregulation, and low mood over time [98]. Consistent with this pathway, clinicians have noted that ongoing sleep disturbance in affected children is often accompanied by crankiness and difficulty concentrating, aligning with a sleep-mediated mechanism for cognitive/behavioral symptoms [99].

Beyond pruritus-driven sleep disruption, additional pathways have been proposed but remain less established: a 2017 study reported altered gut microbiota features in infected children, including increased microbial diversity and higher abundance of *Bifidobacterium*, with some changes persisting after deworming, suggesting that *E. vermicularis* infection and/or treatment may modulate the intestinal ecosystem [100]. Given the recognized role of the gut microbiota in gut–brain and neuroimmune signaling, such microbiome alterations could plausibly contribute to mood or behavior, although direct causal links and inflammatory biomarkers are not yet well defined in this context [100].

Overall, the evidence base is strongest for a sleep-disruption pathway supported by epidemiological associations and clinically coherent symptom patterns, whereas immune- or microbiome-mediated mechanisms remain more speculative and would benefit from longitudinal studies incorporating standardized psychiatric assessments and mechanistic biomarkers before causal inference can be strengthened; nevertheless, clinicians are advised to consider mental health symptoms in children with recurrent enterobiasis, as appropriate antiparasitic treatment may coincide with improvement in sleep and behavioral outcomes [97–100].

#### *Toxocara* spp. (visceral larva migrans)

*Toxocara canis* and *Toxocara cati*, the intestinal roundworms of dogs and cats, are the causative agents of human toxocariasis, a zoonotic infection that is often asymptomatic but may also involve neurological manifestations, with reported associations between toxocariasis and unexplained neurologic symptoms in children [101]. Mechanistically, *Toxocara* larvae can migrate into the central nervous system, leading to neurotoxocariasis. Histopathological and experimental data indicate that this invasion may induce localized neuroinflammation, often characterized by microglial and astrocyte activation (reactive gliosis) and eosinophil-rich granulomatous lesions in the brain [101, 102]. These inflammatory processes may contribute to neuronal injury, demyelination, and disruption of neural circuitry relevant to cognitive function.

In a cross-sectional study of young to middle-aged adults, *Toxocara* seropositivity showed a limited association with selected cognitive test performance; these findings do not establish cognitive dysfunction as an outcome of infection [103]. Population based and cross-sectional studies in older U.S. adults have also reported associations between *Toxocara* seropositivity and lower performance on selected cognitive measures, including domains such as processing speed and attention/working memory; however, these findings do not establish causality or demonstrate a uniform pattern of global cognitive impairment [104, 105]. In addition, review literature has summarized reported neurocognitive and neuropsychiatric manifestations in toxocariasis, but these observations are heterogeneous and should be interpreted cautiously, particularly when inferring treatment-related improvement from limited clinical reports [106].

In an experimental neurotoxocariasis mouse model, Janecek et al. [107] used serial neurobehavioral, sensorimotor, and classic-maze memory assessments and reported reduced exploratory behavior and impaired memory-related performance in infected mice, supporting biological plausibility for neurobehavioral effects in *Toxocara* infection. From a public health perspective, one epidemiological analysis estimated that *Toxocara*-associated cognitive effects in children may contribute substantially to global burden, including disability-adjusted life years (DALYs), although such estimates depend on assumptions and should be interpreted in light of underlying data limitations [108].

Overall, current evidence supports cautious consideration of *Toxocara* infection as a potential contributor to adverse neurocognitive outcomes in some settings, while stronger longitudinal and mechanistic human studies are needed to clarify causality, magnitude of effect, and population-level impact. Preventive measures such as routine pet deworming, environmental hygiene, and reducing children’s exposure to contaminated soil remain important for lowering infection risk in endemic areas.

#### *Schistosoma* (bilharzia)

Schistosomiasis, caused by blood flukes of the genus *Schistosoma*, is a major neglected tropical disease of considerable public health importance [109]. Chronic infection with *Schistosoma* species, also known as bilharzia, contributes to a substantial burden of disease, not only through organ damage but also via psychological and neurological complications. Recent data point to an increased risk of depression among individuals with long-standing schistosomiasis, particularly those with advanced hepatosplenic disease [110].

A 2024 cross-sectional study from China by Hu et al. [111] found that approximately 34% of patients with advanced *Schistosoma japonicum* infection exhibited clinically significant depressive symptoms. In contrast, only about 22% of those with early-stage disease or no infection reported similar symptoms. Depression rates were highest among individuals with hepatosplenomegaly and functional impairment. Multivariate analysis revealed that the most significant predictors of moderate to severe depression in these patients were comorbid serious illnesses and personal economic hardship [111].

Schistosomiasis has been linked to cognitive deficits in endemic populations. For example, a systematic meta-analysis found that infected schoolchildren scored significantly lower on learning and memory tests than uninfected peers [112]. Consistent with this, Gasparotto et al. [113] reported that chronic *S. mansoni* infection in mice impaired spatial learning and memory. Crucially, a recent experiment by Berkiks et al. [114] demonstrated that acute *S. mansoni* infection in adult mice specifically reduced recall memory performance (with *p* < 0.05), even though acquisition and spatial reference memory were unaffected. This suggests that schistosomiasis may selectively impair certain cognitive domains (particularly recall and spatial memory), complementing prior observations of reduced attention and work capacity in chronically infected adults.

Importantly, *Schistosoma* may also affect the brain through direct and indirect mechanisms. Although most eggs lodge in the liver or intestines, in some cases they embolize to the CNS, most often the spinal cord, and less frequently the brain resulting in granulomatous inflammation. Neurological outcomes of cerebral or spinal cord schistosomiasis may include seizures, motor deficits, or paralysis [115].

Moreover, even in the absence of eggs within the CNS, systemic immune responses during chronic infection may impact brain function. Accumulating epidemiological evidence indicates that the more common non-cerebral forms of schistosomiasis are associated with cognitive, learning, memory, and attention deficits in both children and adults [116, 117]. This pattern suggests that the high prevalence of neuropsychiatric manifestations is better explained by systemic inflammation and organ-axis signaling particularly the gut-liver-brain/liver-brain axis than by rare focal mechanisms alone. In chronic schistosomiasis, persistent egg-driven granulomatous inflammation and hepatic fibrosis may sustain cytokine exposure, oxidative stress, and metabolic dysregulation [118, 119], thereby promoting neuroinflammation and behavioral or cognitive changes through pathways analogous to subclinical hepatic encephalopathy.

Systemic infections may promote neuroinflammation and subsequent behavioral or cognitive changes through pathways analogous to subclinical hepatic encephalopathy. Supporting this framework, a murine *S. mansoni* model specifically characterized by systemic infection without direct parasite invasion of CNS demonstrated robust neuroimmune and oxidative stress-related changes in the prefrontal cortex [113]. These findings included astrocyte and microglial activation, oxidative stress signaling, and the accumulation of tau phosphorylation and amyloid-β peptides pathological hallmarks typically associated with idiopathic neurodegenerative diseases [119, 120].

According to Gasparotto [113], these physiological changes were accompanied by impaired spatial learning and memory, as measured by the Morris water maze. Importantly, these phenotypes were attenuated by the administration of praziquantel or an antioxidant regimen (N-acetylcysteine plus deferoxamine), while combined treatment demonstrated a synergistic effect in preventing these alterations. These results support an infection-driven causal pathway linking systemic inflammation and oxidative stress to brain dysfunction. Furthermore, given that hippocampal synaptic plasticity is strongly regulated by brain-derived neurotrophic factor (BDNF), reported alterations in BDNF levels would further strengthen a neuroplasticity-based interpretation of these cognitive outcomes.

Behavioral assessments revealed impaired spatial learning and memory in infected animals. There is also speculation that chronic immune activation in schistosomiasis may disrupt neurotransmitter pathways. Inflammatory cytokines released around egg granulomas may interfere with signaling molecules such as glutamate, serotonin, or dopamine, potentially contributing to mood changes and cognitive impairment [113]. Preclinical studies strengthen biological plausibility by demonstrating that systemic schistosome infection can alter neuroimmune biology and behavior using standard assays under controlled conditions. In a murine *S*,* mansoni* model in which parasites do not invade the CNS, systemic infection induced astrocyte and microglia activation, oxidative damage signaling, tau phosphorylation, and amyloid-β accumulation in the prefrontal cortex, alongside impaired spatial learning and memory measured via the Morris water maze; anthelmintic and antioxidant interventions inhibited many outcomes, supporting a causal infection-driven pathway [113]. Complementing this, a separate study assessed behavior directly and reported increased anxiety-like behavior in infected mice using the open field assay (reduced centre entries) with preserved total distance travelled, arguing against reduced locomotion/sickness as the primary explanation, and also reported altered spatial learning in the Morris water maze [121].

In conclusion, chronic *Schistosoma* infection may contribute to depression and cognitive deficits through a combination of social stressors, long-term illness burden, and neuroimmune mechanisms. In endemic regions, integrating mental health and cognitive assessment, along with appropriate support, into schistosomiasis care may improve both psychological and functional outcomes.

#### *Onchocerca volvulus* and nodding syndrome

Nodding syndrome (NS) is a rare pediatric epileptic encephalopathy reported in specific onchocerciasis-endemic regions of East- Central Africa and is characterized by repetitive atonic head-nodding seizures, often triggered by food intake or cold exposure; affected children may also exhibit progressive cognitive impairment, growth stunting, and malnutrition [122–124].

Epidemiological data consistently show geographic overlap between NS and *Onchocerca volvulus* transmission; for example, a large survey in northern Uganda reported *O. volvulus* positivity in > 93% of children with NS compared with substantially lower prevalence in asymptomatic controls, supporting a strong association at the population level [125].

Importantly for mechanistic interpretation, *O. volvulus* is not considered a typical CNS-invasive parasite adult worms reside in subcutaneous nodules and microfilariae predominantly localize to the skin and eyes suggesting that neurophenotypes are more plausibly mediated by indirect immune mechanisms than by direct parasite presence in brain tissue [125, 126]. A leading hypothesis proposes a para-infectious autoimmune process involving molecular mimicry, whereby immune responses to *O. volvulus* antigens may cross-react with neuronal targets, contributing to neuroinflammation and seizures [126]. Supporting this, Johnson et al. reported elevated autoantibodies against leiomodin-1 in children with NS, with cross reactivity to *O. volvulus* antigens and neurotoxic effects in vitro, although subsequent studies have yielded mixed findings and not all patients demonstrate these antibodies, indicating heterogeneity and/or additional contributing factors [127]. Other proposed pathways include chronic microglial activation, sustained pro-inflammatory cytokine signaling, and the modifying roles of malnutrition or co-infections, while the occurrence of high *O. volvulus* transmission in some regions without NS suggests that environmental or genetic cofactors may influence susceptibility and clinical expression [127, 128].

Beyond NS, increased epilepsy prevalence has been reported in onchocerciasis-endemic areas even outside classic NS phenotypes, implying that *O. volvulus* exposure may broadly elevate seizure risk through immune-mediated pathways, although causality and the specific mechanistic mediators remain incompletely resolved [130]. Overall, evidence is strong for epidemiological association and plausible immune-mediated mechanisms, but key limitations include inconsistent biomarker replication (e.g., leiomodin-1 antibodies), likely multifactorial causation, and limited longitudinal mechanistic studies that integrate standardized neurocognitive assessment with immune profiling and cofactor measurement [127–130].

### Post-malaria neuropsychiatric sequelae (cerebral malaria)

Severe malaria, particularly cerebral malaria (CM) due to *Plasmodium falciparum*, is increasingly recognized as a condition with important long-term neurological and psychological sequelae among survivors, especially children, even as acute case fatality has declined with improved management [131].

In mechanistic terms, CM is defined by parasite-infected erythrocyte sequestration within the brain microvasculature, driving endothelial activation, inflammatory cytokine release (notably TNF-α), blood-brain barrier disruption, cerebral swelling, and downstream neural injury, whereas severe malarial anemia represents a non-comatose severe phenotype that may still affect neurodevelopment through systemic and inflammatory stressors [132, 133].

Clinically, 20–30% of children surviving CM exhibit persistent cognitive and/or behavioral deficits [131]; for example, in a prospective Ugandan cohort, 21.4% of CM survivors had significant impairment at six months in domains including attention, memory, and non-verbal reasoning compared with 5.8% of community controls, and adjusted analyses indicated that CM survivors were 3.7 times more likely to demonstrate measurable cognitive impairment, often manifesting as deficits in working memory, executive attention, and learning with implications for school performance [134]. Emotional and behavioral effects have also been documented: in a two-year longitudinal Ugandan study, children who survived CM or severe malarial anemia showed higher internalizing symptoms (e.g., anxiety, withdrawn/depressed behavior) and externalizing problems (e.g., aggression, hyperactivity) than community controls, with differences persisting at 24 months, and importantly, increased behavioral difficulties were also seen after severe malarial anemia without coma, supporting the relevance of non-cerebral severe malaria to neurodevelopmental outcomes [135, 136]. The likelihood and severity of sequelae correlate with acute disease severity, as prolonged coma and multiple seizures during CM increase risk; according to Boivin [134] children with later cognitive impairment had experienced more seizures (mean ≈ 4) and longer coma duration, implicating acute brain insults such as seizure-related excitotoxicity, hypoxia, and intracranial hypertension as contributors to persistent dysfunction [134, 136]. Autopsy and neuroimaging studies frequently demonstrate diffuse brain edema, microhemorrhages, and white-matter injury, and intense neuroinflammation may promote neuronal apoptosis in vulnerable regions including the hippocampus and cortex, providing biological plausibility for learning and memory deficits [137]. Additional post-infectious phenomena are also reported, including post-malaria neurological syndrome with delayed-onset cognitive impairment, seizures, or neuropsychiatric symptoms weeks to months after recovery suggesting prolonged or secondary immune-mediated effects and an increased risk of subsequent epilepsy in a subset of survivors, potentially related to gliotic scarring and focal injury [138, 139].

Overall, the evidence base is strong because it includes prospective and longitudinal human cohorts and supportive neuropathological/neuroimaging data, but key limitations include heterogeneity in severity phenotypes, variability in neurocognitive assessment tools across settings, and the need for more standardized long-term psychiatric phenotyping and biomarker-linked prognostic studies to better distinguish direct CNS injury from systemic inflammatory contributions [131–139].

A comparative summary of neuropsychiatric manifestations, proposed mechanisms, and the strength of available evidence across selected parasitic infections is presented in Table 1.

**Table 1:** Comparative summary of neuropsychiatric manifestations, proposed mechanisms, and evidence strength across selected parasitic infections

Parasite / Diseases	Human neuropsychiatric/cognitive observations	Human evidence type	Experimental behavioral data	Direct CNS invasion necessary?	Proposed mechanism(s)	Mechanistic support	Overall evidence strength	Key gaps / limitations	Refs.
*Taenia solium* (Neurocysticercosis, NCC)	Seizures, headache, cognitive impairment, confusion, dementia, depression, anxiety, hallucinations, personality/behavioral changes (varies by cyst burden/location and host response)	Clinical case observations and epidemiological data in endemic regions (Latin America, Africa, Asia).	Not provided	Yes (for NCC)	Direct CNS cyst localization with secondary neuroinflammation, BBB disruption, immune-cell infiltration, intracranial pressure effects; lesion-location effects on behavior/cognition	Symptom expression is shaped by cyst number, size, anatomical location (e.g., frontal lobe), and host immune responsiveness.	Strong (for direct CNS/inflammatory pathway in NCC)	Lesion heterogeneity, limited standardized psychiatric assessment, few longitudinal mechanistic studies	[72–75]
*Toxoplasma gondii* (Toxoplasmosis/latent toxoplasmosis)	Encephalitis, chorioretinitis, fetal brain damage (severe forms); in chronic/latent infection: associations with schizophrenia, mood/anxiety disorders, altered personality traits, and risk-taking behavior	Epidemiological and clinical association studies; experimental/animal studies	Yes, rodent studies show anxiety-like behavior (EPM, OFT), with neuroinflammatory and neurotransmitter changes; microbiota-transfer evidence supports a gut-mediated component	Not always (severe CNS disease involves direct invasion; chronic neuropsychiatric effects may also involve indirect gut–brain/immune pathways)	Neuroinflammation, gut dysbiosis/gut–brain axis, microglial activation, cytokine signaling, serotonergic changes (5-HT), and direct neurotransmitter modulation (dopamine/L-DOPA, glutamatergic/GABAergic pathways)	Parasite-encoded AAH1/AAH2 enzymes produce L-DOPA; antibiotic-mediated microbiota depletion attenuates behavioral changes; fecal microbiota transfer (FMT) exacerbates reduced exploration; lower 5-hydroxytryptamine (serotonin)	Human: Association-based (not causal); modest and heterogeneous. Experimental: Strong mechanistic support for neuroinflammation and behavioral changes, though highly model-dependent.	Difficulty distinguishing “sickness behavior” from specific psychiatric phenotypes; lack of evidence that parasite enzymes universally elevate host brain dopamine; mixed results for mood/anxiety outcomes in humans.	[18], [19], [59], [80–89]
American trypanosomiasis / Chagas disease*(Trypanosoma cruzi)*	Cognitive impairment (memory and executive function); symptoms can occur without overt cardiac pathology.	Reports in chronic patients; association with systemic biomarkers.	Not provided	No (typically indirect in chronic phase); invasion occurs mainly in acute/immuno-compromised cases.	Chronic systemic immune activation; neuroendocrine dysregulation; indirect organ-axis effects (gut-brain axis).	Elevated proinflammatory cytokines and stress hormones; enteric nervous system damage (megaesophagus/megacolon).	Moderate; biologically plausible but requires more longitudinal data.	Clinical heterogeneity; potential confounding factors; lack of longitudinal studies linking biomarkers to neurocognition.	[90–92]
*Trypanosoma brucei* (African trypanosomiasis / sleeping sickness)	Sleep–wake disruption (insomnia/somnolence), cognitive dysfunction, apathy, personality change, mania, aggression, hallucinations, and suicidal ideation.	Clinical staging studies, neuroclinical observations, CSF biomarker studies	Not provided	Yes, crossing the blood–brain barrier (BBB) marks the transition to the late encephalitic stage.	CNS inflammation; activation of astrocytes and microglia; proinflammatory cytokine cascade; endothelial/neuronal damage via variant surface glycoproteins.	Elevated IL-6 and IL-10 in cerebrospinal fluid (CSF); parasite proliferation in CSF and brain parenchyma.	Strong; supported by clear clinical staging and consistent CSF biomarkers.	Heterogeneity by disease stage; limited use of standardized psychiatric phenotyping.	[73], [84], [90], [93–96]
*Enterobius vermicularis* (Enterobiasis / pinworm)	Anxiety, depressive symptoms/low mood, sleep disturbance/insomnia, irritability, mood instability, fatigue, concentration difficulties (mainly in children)	Nationwide cohort study + case reports/clinical observations	Not provided	No	Sleep disruption secondary to nocturnal pruritus (most supported); possible gut microbiome and neuroimmune/gut–brain axis involvement (speculative)	Significant odds ratios for sleep and mood disorders in large cohorts; evidence of altered gut microbiota (increased diversity and *Bifidobacterium* abundance).	Moderate (clinical association/sleep pathway); Limited for gut–brain mechanism	Observational confounding, reliance on pediatric data, limited mechanistic biomarkers, few longitudinal/interventional psychiatric outcome studies	[97–100]
*Toxocara* spp. (Toxocariasis / visceral larva migrans; neurotoxocariasis)	Cognitive deficits (working memory, attention, processing speed), lower IQ/academic performance, learning difficulties; behavioral changes/depressed mood reported in some cases	Population-based seroepidemiology, case-control studies, clinical cases	Yes, rodent studies report memory impairment and reduced exploratory behavior	Not always (cognitive associations may be seen with exposure/seropositivity); Yes in neurotoxocariasis	CNS larval migration (neurotoxocariasis) with neuroinflammation, reactive gliosis (microglia/astrocytes), eosinophilic granulomas, neuronal injury/demyelination; possible broader inflammatory effects	Histopathological data showing neuronal injury, demyelination, and disrupted neural circuitry; experimental sensorimotor and maze assessments.	Limited/Cautious: Supports biological plausibility and population-level association, but does not establish uniform global impairment.	Seropositivity does not confirm active CNS infection; residual confounding possible; limited longitudinal and standardized neuropsychiatric assessments	[101–108]
*Schistosoma* spp. (Schistosomiasis / bilharzia)	Depression (especially in advanced hepatosplenic disease), cognitive/learning/memory/attention deficits; rare cerebral/spinal schistosomiasis may cause seizures and motor deficits/paralysis	Cross-sectional studies, epidemiological studies, meta-analyses, clinical reports; animal studies	Yes, murine *S. mansoni* studies show impaired spatial learning/memory (Morris water maze) and anxiety-like behavior (open field), with preserved locomotion in some models	No for common neuropsychiatric/cognitive effects; Yes in rare neuroschistosomiasis	Systemic inflammation and organ-axis signaling (gut–liver–brain/liver–brain), chronic hepatic fibrosis/granulomatous inflammation, cytokine exposure, oxidative stress, neuroinflammation; rare direct CNS egg embolization/granulomatous lesions	Astrocyte/microglial activation, tau phosphorylation, and amyloid-β accumulation in the prefrontal cortex; altered BDNF levels; cytokine interference with glutamate, serotonin, or dopamine.	Strong biological plausibility: Experimental data shows “rescue” of phenotypes with praziquantel and antioxidants (N-acetylcysteine + deferoxamine).	Human mechanistic biomarkers and longitudinal studies are limited; social/economic confounding for depression; heterogeneity by species/stage and organ involvement	[110–121]
*Onchocerca volvulus* (Onchocerciasis-associated epilepsy / Nodding syndrome)	Nodding seizures (atonic head nodding), progressive cognitive decline, epilepsy, growth stunting, malnutrition; broader increased epilepsy burden in endemic areas	Epidemiological studies, endemic-region surveys, case-control/clinical studies, immunologic studies	Not provided	No (parasite does not typically invade CNS)	Immune-mediated/para-infectious mechanisms, molecular mimicry (e.g., anti–leiomodin-1 autoantibodies), neuroinflammation, microglial activation, cytokine signaling; possible roles of malnutrition/co-infections and environmental/genetic cofactors	> 93% *O. volvulus* positivity in NS cases; discovery of elevated leiomodin-1 autoantibodies that cross-react with parasite antigens.	Strong epidemiological link, but the specific mechanistic biomarkers (leiomodin-1) show inconsistent replication across studies.	Multifactorial nature (role of malnutrition/co-infections); inconsistent antibody findings; lack of longitudinal studies integrating immune profiling with neurocognitive testing.	[122–130]
*Plasmodium falciparum* (Cerebral malaria / post-malaria neuropsychiatric sequelae)	Persistent cognitive deficits (attention, memory, non-verbal reasoning, learning/executive difficulties), behavioral/emotional problems (internalizing/externalizing symptoms), epilepsy, delayed post-malaria neuropsychiatric/neurological syndrome	Prospective cohorts, longitudinal studies, neuroimaging/autopsy studies, clinical follow-up studies	Not provided	No direct parasite invasion of neurons required (pathology driven by microvascular sequestration, inflammation, BBB disruption, edema, secondary brain injury)	Cerebral microvascular sequestration, cytokine-driven neuroinflammation (e.g., TNF-α), BBB disruption, cerebral edema, hypoxia/excitotoxicity/seizure-related injury, neuronal apoptosis, gliosis; delayed immune-mediated effects	Strong for clinical association and biological plausibility (human longitudinal and pathology data)	Correlation between coma duration/seizure frequency and impairment; imaging showing diffuse brain edema, microhemorrhages, and white-matter injury; gliotic scarring.	Heterogeneity by severity, treatment timing, and age; limited standardized long-term psychiatric phenotyping in some settings; need more biomarker-linked prognostic studies	[131–139]

### Regional disparities in parasite burden and neuropsychiatric comorbidities

High parasite prevalence across Africa, Asia, and Latin America often affecting more than 20–30% of the population coincides with a substantial burden of neuropsychiatric comorbidities. Globally, an estimated 1.5 billion people, or approximately 24% of the world’s population, are infected with soil-transmitted helminths alone. These infections are especially concentrated in sub-Saharan Africa, South and Southeast Asia, and Latin America, where warm climates, inadequate sanitation, and poverty perpetuate transmission [140]. According to the World Health Organization, intestinal worm infections disproportionately impact the poorest and most deprived communities in tropical regions. In parallel, mental health concerns associated with chronic parasitic infections are gaining increasing attention.

In Africa, a systematic review by Lampard- Scotford et al. [141] revealed that individuals with parasitic infections had significantly higher rates of psychiatric disorders (58.2%) compared to uninfected individuals (41.8%). The analysis showed that the odds of having a mental illness were approximately four times higher in parasite-positive individuals, indicating a strong epidemiological association. Notably, the reviewed studies implicated both helminthic and protozoan infections in mood, anxiety, and stress-related disorders.

Similarly, many regions in Asia continue to endure endemic parasitic diseases alongside rising mental health burdens. Although mass deworming programs have led to recent declines in soil-transmitted helminths such as hookworm, roundworm, and whipworm [142, 143], protozoan infections remain widespread. For instance, *Toxoplasma gondii* seroprevalence in Asia varies widely, from approximately 13% to as high as 85% in certain communities [144]. Recent meta-analyses suggest that psychiatric patients in low-income Asian settings experience particularly high rates of parasitic infections. Abdoli et al. [145] reported that approximately 25% of psychiatric patients globally show evidence of protozoan infection, with Asia exhibiting the highest regional prevalence. These trends may be exacerbated by high population density, gaps in sanitation infrastructure, and co-infection with multiple parasites.

In Latin America and the Caribbean, the parasitic disease burden is shaped by socio-economic disparities and the presence of neurotropic parasites that directly affect the nervous system. While the prevalence of soil-transmitted helminths has declined in some areas, high rates persist in impoverished regions such as rural Amazonia and parts of the Andes [146, 147]. Moreover, chronic parasitic infections in Latin America are strongly linked to psychiatric morbidity. A meta-analysis by Daré et al. [148] found that approximately 45% of patients with either Chagas disease or neurocysticercosis exhibited comorbid anxiety or depression. Latent *T*. *gondii* infections are also highly prevalent in parts of Latin America, particularly in Brazil, and have been associated with an increased risk of psychotic disorders. Daré et al. [148] further observed that exposure to *T. gondii* or *Toxocara* was associated with more than double the odds of schizophrenia or bipolar disorder in populations from developing countries (pooled OR ≈ 2.3). These findings support the hypothesis that *T*. *gondii* infection may act as a contributory factor in the development of schizophrenia.

In summary, parasitic infections remain endemic across large segments of Africa, Asia, and Latin America. A growing body of epidemiological evidence indicates that these infections frequently coexist with and may contribute to neuropsychiatric morbidity at the population level. This underscores the need for integrated approaches that consider both infectious and mental health dimensions in public health interventions, particularly in low- and middle-income countries.

### Malnutrition, poverty, and polyparasitism as interacting determinants

The impact of parasitic infections on the gut–brain axis is shaped by a constellation of interacting factors, particularly malnutrition, poverty, and polyparasitism. These determinants are frequently interwoven and prevalent in regions where parasitic diseases are endemic, collectively amplifying the neurological and developmental consequences of infection [149, 150].

Malnutrition and parasitic infections are mutually reinforcing, forming a detrimental cycle that undermines both physical and cognitive development [151]. Intestinal parasites impair nutritional status through multiple mechanisms, they consume host nutrients and blood, induce malabsorption, and cause chronic diarrhea that depletes essential micronutrients [152]. For instance, persistent infections with *Ancylostoma duodenale* or *Trichuris trichiura* are commonly associated with iron-deficiency anemia and growth stunting in children. Nutrient deficiencies, especially during critical periods of early development can result in long-term cognitive deficits, affecting memory, attention, and academic performance [153]. *Giardia duodenalis*, a protozoan that causes prolonged diarrhea, has been linked to impaired cognitive outcomes in children, primarily through zinc and iron deficiencies and related oxidative stress [154]. Moreover, malnourished individuals are more susceptible to severe parasitic infections due to compromised immune function [155]. This bidirectional relationship creates a self-perpetuating loop wherein malnutrition and infection together exacerbate neurodevelopmental harm. Importantly, field studies have demonstrated that interventions combining deworming with nutritional supplementation can lead to measurable improvements in cognitive scores among school-aged children, underscoring the critical role of nutrition in modulating parasitic disease outcomes [156, 157].

Parasitic infections are deeply embedded within the broader context of poverty and its associated social determinants. Impoverished communities frequently lack access to clean water, adequate sanitation, and healthcare infrastructure, creating conditions conducive to the transmission of multiple parasitic species [158, 159]. Poverty also contributes to chronic psychosocial stress and reduced educational opportunities, both of which can negatively affect mental health. When layered with the physiological burden of chronic infections, these stressors compound the neuropsychiatric toll. As noted by the WHO, helminth infections disproportionately affect the poorest communities, those with the least access to hygiene, sanitation, and healthcare services [160]. Within these contexts, children bearing heavy parasitic burdens often perform poorly in school, limiting their future socio-economic mobility and perpetuating cycles of poverty, illness, and cognitive disadvantage.

**Fig. 3:**
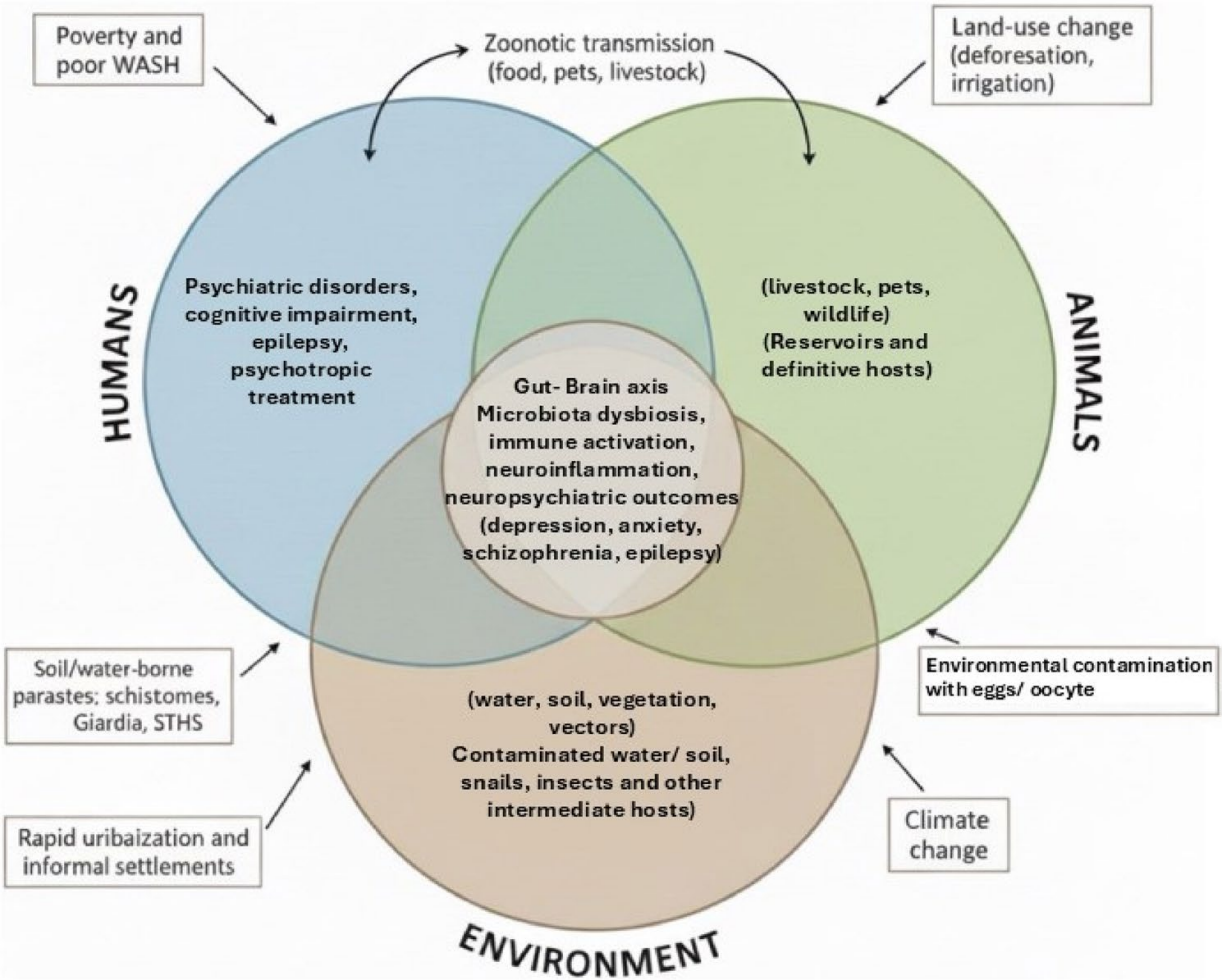
One Health conceptual model linking parasitic infections to mental health via the microbiota–immune–brain axis

Polyparasitism is common in many endemic areas, where a single person can be infected at the same time with several different parasitic species. These co-infections can have harmful effects on the host. Different parasites may target distinct organs or deplete specific nutrients, leading to compounded immune dysregulation and nutritional deficiencies [161]. For example, research in the Brazilian Amazon by Jardim-Botelho et al. [162], has shown that children with multiple concurrent infections (e.g., *Ascaris lumbricoides* and *Ancylostoma duodenale*) score significantly lower on cognitive assessments compared to uninfected or singly infected peers. Co-infections also complicate immune responses; helminths, for instance, often shift immunity toward a regulatory or Th2-dominant profile, which may impair resistance to intracellular pathogens like protozoa [163]. This interplay between immune modulation, nutritional depletion, and multiple infections contributes to a cumulative pathogenic burden on the gut–brain axis. The resulting cycle of polyparasitism, malnutrition, and developmental delay underscores the need for integrated, multi-pathogen control strategies rather than isolated interventions targeting single species. Figure 3 illustrates how humans, animals, and the environment intersect to shape exposure to parasitic infections and downstream neuropsychiatric outcomes.

## Conclusion

In conclusion, converging clinical and experimental evidence indicates that diverse parasitic infections can promote sustained immune-to-brain signaling that culminates in neuroinflammation and measurable behavioral dysfunction. A key emerging insight is that neuroinflammatory outcomes are not restricted to classically neurotropic parasites; intestinal and tissue-dwelling helminths may also influence the CNS by reshaping the gut microbiota, increasing barrier permeability, and altering systemic cytokine tone, thereby priming microglia and astrocytes and disrupting blood–brain barrier integrity. Downstream effects on neurotransmitter systems and infection-associated transcriptional remodeling via kynurenine-pathway activation, microRNA regulation, and epigenetic mechanisms provide plausible routes to persistent cognitive and affective phenotypes even after acute infection resolves. Future work should prioritize harmonized longitudinal human cohorts, mechanistically rigorous gnotobiotic and metabolomics approaches, and careful separation of parasite, microbiota and immune-mediated effects. Identifying shared neuroinflammatory nodes across parasitic diseases will be essential for developing tractable biomarkers and host directed therapies to reduce long-term neuropsychiatric risk.

## Data Availability

The datasets supporting the conclusions of this article are included within the article.

## Abbreviations

CNS: Central nervous system
TLR: Toll-like receptor
IL-: Interleukin-
IL-1β: Interleukin-1 beta
TNF-α: Tumor necrosis factor alpha
NF-κB: Nuclear factor kappa-light-chain-enhancer of activated B cells
AaaH: Aromatic amino acid hydroxylase
EPM: Elevated plus maze
FST: Forced swim test
TST: Tail suspension test
L-DOPA: Levodopa (L-3,4-dihydroxyphenylalanine)
DBH: Dopamine beta-hydroxylase
CpG methylation: Cytosine-phosphate-guanine dinucleotide methylation
lncRNA: Long non-coding RNA
miRNA: MicroRNA
NODs: Nucleotide-binding oligomerization domain-containing proteins
MyD88: Myeloid differentiation primary response 88
CD8⁺: Cluster of differentiation 8–positive T lymphocytes
IFN-γ: Interferon-gamma
Th1: T helper 1 lymphocyte subset
Th2: T helper 2 lymphocyte subset
IgE: Immunoglobulin E
ICAM-1: Intercellular adhesion molecule 1
VCAM-1: Vascular cell adhesion molecule 1
BBB: Blood–brain barrier
CXCL10: C-X-C motif chemokine ligand 10 (IP-10)
GM-CSF: Granulocyte–macrophage colony-stimulating factor
NO: Nitric oxide
OFT: Open field test
ROS: Reactive oxygen species
LPS: Lipopolysaccharide
IDO: Indoleamine 2,3-dioxygenase
GABA: Gamma-aminobutyric acid
TGF-β: Transforming growth factor beta
IgA: Immunoglobulin A
SCFAs: Short-chain fatty acids
BhTnaA: Blastocystis tryptophanase A
NCC: Neurocysticercosis
DALYs: Disability-adjusted life years
NS: Nodding syndrome
CM: Cerebral malaria
PTSD: Post-traumatic stress disorder
WHO: World Health Organization
